# Complete genome sequence of the type strain *Piscinibacterium candidicorallinum* LMG 29480

**DOI:** 10.1128/mra.00139-25

**Published:** 2025-04-07

**Authors:** Debajyoti Deb, William M. Moe

**Affiliations:** 1 Department of Civil and Environmental Engineering, Louisiana State University 5779, Baton Rouge, Louisiana, USA; Indiana University Bloomington, Bloomington, Indiana, USA

**Keywords:** *Piscinibacterium*, *Burkholderiales*, nitrogen metabolism, polyhydroxyalkanoate synthesis, Pseudomonadota, *Burkholderiaceae*

## Abstract

*Piscinibacterium candidicorallinum* LMG 29480 is a motile gram-stain-negative bacterium able to reduce nitrate and accumulate poly-β-hydroxybutyrate storage granules. Here, we report the complete genome sequence (3,829,664 bp, 65.16 mol% G+C content), which may prove useful in future taxonomic comparisons and efforts to assess the role of *Piscinibacterium* in nutrient cycling.

## ANNOUNCEMENT

*Piscinibacterium candidicorallinum* LYH-15^T^ (=LMG 29480, = KCTC 52168), type strain of the species, was isolated from a freshwater fish pond in Taiwan (1). It is a motile, gram-stain-negative, rod-shaped bacterium with an ability to reduce nitrate and accumulate poly-β-hydroxybutyrate granules (1). Based on 16S rRNA gene sequencing, related bacteria have been reported in wastewater treatment systems employing biological nitrogen removal (2). A draft genome sequence for *Piscinibacterium candidicorallinum* KCTC 52168 was recently reported (GCA_042646225.1); however, the assembly based on only Illumina sequencing was fragmented (23 contigs in 13 scaffolds). Here, we report the complete genome sequence for the *Piscinibacterium candidicorallinum* type strain.

*Piscinibacterium candidicorallinum* LMG 29480 was obtained from the BCCM/LMG culture collection (Ghent, Belgium). Cells grown in R2A broth (12 days, 25℃) were harvested by centrifugation (30 min, 4°C, 3,000 × *g*), followed by DNA extraction using a GenElute Bacterial Genomic DNA Kit (Sigma-Aldrich). Sequencing involved a combination of long- and short-read platforms. Long-read sequencing library preparation employed 1.5  µg high-molecular-weight DNA and an Oxford Nanopore Technologies (ONT) ligation sequencing kit (SQK-LSK114). There was no intentional shearing or size selection prior to library preparation. The library was sequenced at EzBiome (Gaithersburg, MD) on an ONT MinION Mk1C device with R10.4.1 flow cell (3). Dorado v 7.4.12 (ONT) was used for base calling using the SUP model and adapter/barcode removal. Fitlong v 0.2.1 (github.com/rrwick/Filtlong) was used to deplete the initial library (151,362 reads) of reads with the lowest 5% of fastq scores. *De novo* assembly of filtered Nanopore data (*N*_50_ = 8,568 bp) was performed using –nano-hq assembly mode in the Flye v 2.9.5 pipeline (4).

Short-read sequencing library construction employed a NEBNext Ultra Library Prep Kit for Illumina. Paired-end sequencing (2 × 150  bp) performed using a NextSeq 2000 system (Illumina) resulted in 3,443,506 reads (total length 1.095 Gb). BBDuk in the BBMap v. 39.13 package (sourceforge.net/projects/bbmap/) was used to trim and filter low-quality reads (bbduk.sh -Xmx1g ref = bbmap/resources/adapters.fa threads = 10 ktrim = r k = 23 mink = 11 hdist = 1 qtrim = r tpe tbo). Filtered Illumina data were used in Polypolish v 0.6.0 (5) to polish the ONT assembly. Circularity was assessed using Flye v 2.9.5. The genome was rotated to start of the *dnaA* gene as inferred by (6) prior to annotation using NCBI Prokaryotic Genome Annotation Pipeline v 6.9 (7). For all software, default parameters were used except where otherwise noted.

The completed genome sequence of *P. candidicorallinum* LMG 29480 is comprised of a single, circular chromosome of 3,829,664 bp (coverage 99.8× ONT, 262× Illumina), with 65.16 mol% G+C content. Completeness estimated via CheckM v 1.4.0 (8) with the *Betaproteobacteria* marker set was 99.36%. The 3,472 predicted genes include 66 tRNAs and single-copy 5S, 16S, and 23S rRNA genes. The 16S rRNA gene in the genome assembly is identical to the sequence reported previously (LT158233) (1). Average nucleotide identity (ANI) between the closed genome (CP180191) and the earlier (fragmented) assembly (GCA_042646225.1) calculated using FastANI (9) v 1.34 was 99.9931%. Consistent with experimental observations that the strain can reduce nitrate (1), the genome harbors several genes associated with nitrate and nitrite transmembrane transport and reduction.

## Data Availability

Sample information, sequence, and genomic assembly/annotation are accessible under the NCBI BioProject, BioSample, and whole-genome sequence accession numbers PRJNA1216302, SAMN46432207, and CP180191, respectively. The Oxford Nanopore and Illumina sequencing reads are available from the NCBI Sequence Read Archive (SRA) under accession numbers SRR32150714 and SRR32150713, respectively.
